# Induction of apoptosis and G_2_/M arrest by ampelopsin E from *Dryobalanops* towards triple negative breast cancer cells, MDA-MB-231

**DOI:** 10.1186/s12906-016-1328-1

**Published:** 2016-09-08

**Authors:** Napsiah Abd Rahman, Latifah Saiful Yazan, Agustono Wibowo, Norizan Ahmat, Jhi Biau Foo, Yin Sim Tor, Swee Kong Yeap, Zainal Abidin Razali, Yong Sze Ong, Sharida Fakurazi

**Affiliations:** 1 Laboratory of Molecular Biomedicine, Institute of Bioscience, Universiti Putra Malaysia, 43400 UPM Serdang, Selangor Malaysia; 2 Department of Biomedical Science, Faculty of Medicine and Health Sciences, Universiti Putra Malaysia, 43400 UPM Serdang, Selangor Malaysia; 3 Faculty of Applied Sciences, Universiti Teknologi MARA, 40450 Shah Alam, Selangor Malaysia; 4 Laboratory of Vaccines and Immunotherapeutics, Institute of Bioscience, Universiti Putra Malaysia, 43400 UPM Serdang, Selangor Malaysia

**Keywords:** *Dryobalanops sp*, Ampelopsin E, Cytotoxicity, Apoptosis, Cell cycle arrest

## Abstract

**Background:**

Several compounds isolated from *Dryobalanops* have been reported to exhibit cytotoxic effects to several cancer cell lines. This study investigated the cytotoxic effects, cell cycle arrest and mode of cell death in ampelopsin E-treated triple negative cells, MDA-MB-231.

**Methods:**

Cytotoxicity of ampelopsin E, ampelopsin F, flexuosol A, laevifonol, Malaysianol A, Malaysianol D and nepalensinol E isolated from *Dryobalanops* towards human colon cancer HT-29, breast cancer MDA-MB-231 and MCF-7, alveolar carcinoma HeLa and mouse embryonic fibroblast NIH/3 T3 cells were determined by MTT assay. The cells were treated with the compounds (0.94–30 μM) for 72 h. The mode of cell death was evaluated by using an inverted light microscope and annexin V/PI analysis. Cell cycle analysis was performed by using a flow cytometer.

**Results:**

Data showed that ampelopsin E was most cytotoxic toward MDA-MB-231 with the IC_50_ (50 % inhibition of cell viability compared to control) of 14.5 ± 0.71 μM at 72 h. Cell shrinkage, membrane blebbing and formation apoptotic bodies characteristic of apoptosis were observed following treatment with ampelopsin E. The annexin V/PI flow cytometric analysis further confirmed that ampelopsin E induced apoptosis in MDA-MB-231 cells. Cell cycle analysis revealed that ampelopsin E induced G_2_/M phase cell cycle arrest in the cells.

**Conclusion:**

Ampelopsin E induced apoptosis and cell cycle arrest in MDA-MB-231 cells. Therefore, ampelopsin E has the potential to be developed into an anticancer agent for treatment of triple negative breast cancer.

## Background

Cancer is a leading cause of mortality and morbidity worldwide over the years. Approximately, 14.1 million cancer cases and 8.2 million cancer-related deaths worldwide were recorded in 2012. Breast cancer ranked as second most commonly diagnosed cancer (1.67 million cases) after lung cancer (1.82 million cases) [6]. Breast cancer represents a heterogeneous group of tumors with several characterizations based on their morphological and biological features, behavior and response to treatments [9]. It can be classified into different subgroups by the expression of estrogen receptor (ER), progesterone receptor (PR) and human epidermal growth factor receptor 2 (HER2). Among all the breast cancers cases, 25 to 30 % of them are ER negative or triple negative breast cancer (TNBC) known to be most aggressive with high metastatic potential [17] especially to the vital organs such as brain and lungs. Approximately, over 80 % of hereditary *BRCA1* mutation is TNBC [21]. Chemotherapy is the common treatment for TNBC patients (the use of taxanes, ixabepilones, anthracyclines, platinum agents, biologic agents and anti-EGFR drugs) [13]. However, the treatment comes with adverse effects including multidrug resistance and congestive heart failure [2]. Therefore, new drug for management of TNBC is in great demand.

Natural products play an important role in cancer research. There are about more than two third of the currently available anticancer agents are derived from natural products between 1940s to 2006 [29]. The *Dryobalanops* or locally called as Kapur from *Dipterocarpaceae* family can only be found in the tropical forest of Malesia such as Peninsular Malaysia, Sumatra and Borneo [3]. It is very unique with only seven species worldwide including *D. rappa, D. aromatica, D. lanceolata, D. beccarii, D. fusca, D. keithii* and *D. oblongifolia.* Approximately 200 oligostilbenoid constituents have been isolated from *Dipterocarpacea* family [15]. The uniqueness and complexity of the structure of oligostilbenoid in each genera has attracted scientists from various fields to investigate its phytochemical constituents, bioactivities, biogenesis and chemotaxonomy [34]. There are several types of oligostilbenoid constituents including ampelopsin E [27], flexuosol A [22] and Malaysianol D [34] found in *D. beccarii*. Malaysianol A was successfully isolated from *D. aromatica* [33], while nepalensinol E [35], ampelopsin F [31] and laevifonol [11] can be found mostly in all *Dryobalanops sp*. Oligostilbenoid constituents were reported to possess some bioactivities such as anti-diabetogenic effect [24] and anti-angiogenesis [18]. In the previous study, Wibowo et al. [34] reported that ampelopsin E was the most cytotoxic towards breast adenocarcinoma cells (MCF-7). However, there is no yet any study on the mechanisms underlying the cytotoxicity of oligostilbenoid compounds especially ampelopsin E towards cancer cell lines. This study investigated the cytotoxic effects, cell cycle arrest and mode of cell death involved in ampelopsin E-treated triple negative cells, MDA-MB-231.

## Methods

### Compounds

Pure compounds of *Dryobalanops* (*Dryobalanops aromatica* Gaertn, *Dryobalanops beccarii* Dyer and *Dryobalanops lanceolata* Burck and *Dryobalanops rappa* Becc) which are ampelopsin E, ampelopsin F, flexuosol A, laevifonol, Malaysianol A, Malaysianol D and nepalensinol E, were kindly supplied by the Faculty of Applied Sciences, Universiti Teknologi MARA, Shah Alam, Selangor, Malaysia.

### Reagents and chemicals

RPMI-1640 without phenol red (Roswell Park Memorial Institute Medium) was purchased from Nacalai Tesque Inc (Kyoto, Japan). DMEM-F12 (Dulbeco’s Modified Eagle Medium), epidermal growth factor (EGF), hydrocortisone, cholera toxin, insulin, trypan blue solution, MTT powder, propidium iodide (PI) and RNase A were purchased from Sigma-Aldrich, St. Louis, MO, USA. Penicillin-streptomycin antibiotic and trypsin-EDTA were purchased from PAA Laboratories (Pasching, Austria). MycoplexTM foetal bovine serum (FBS) was purchased from Commerce Ave (California, USA).

### Cell culture

The dependent-hormonal breast adenocarcinoma (MCF-7), independent-hormonal breast adenocarcinoma (MDA-MB-231), human colon adenocarcinoma (HT29), alveolar carcinoma (A-549), cervical adenocarcinoma (HeLa), mouse embryonic fibroblast (NIH/3 T3) and normal breast epithelial (MCF-10A) cell lines were purchased from the American Type and Culture Collection (ATCC), USA. All the cell lines except MCF-10A were cultured in RPMI-1640 medium supplemented with 10 % foetal bovine serum (FBS) and 1 % antibiotics (100 IU/mL penicillin and 100 μg/mL streptomycin) and maintained in a 37 °C incubator with humidified atmosphere of 5 % CO_2_. MCF-10A cells were cultured in DMEM-F12 medium supplemented with 10 % foetal bovine serum, 0.5 mg/mL hydrocortisone, 10 μg/mL insulin, 100 ng/mL cholera toxin and 20 ng/mL epidermal growth factor.

### Determination of cytotoxicity

The cytotoxic effects of the compounds were evaluated by the MTT [3-(4,5-dimethylthiazol-2-yl)-2,5-diphenyltetrazolium bromide] assay [25]. The cells (50,000 cells/mL) were treated with various concentrations of the compounds (0.94–30 μM) in a 96-well plate for 72 h. Untreated controls were also included. Following incubation, 20 μL of 5 mg/mL MTT solution was added to each well and incubated at 37 °C for 3 h. Next, 100 μL of DMSO was added to dissolve the purple precipitate of formazan crystal. The absorbance at 570 nm and 630 nm as reference wavelength was measured by a microplate reader (BioTek EL 800, United States). The IC_50_ (concentration of the compound that will cause 50 % inhibition of cell growth compared to the control [1]) was obtained from the fit standard curve of percentage cell viability on the ordinate against the compound concentration on the abscissa:

Cell growth inhibition (%) = [1 - (ODTreated/OD Control)] × 100.

Based on the IC_50_ value, the most potent compound was subjected to further analysis.

### Determination of morphological changes

Briefly, 150,000 cells per well of MDA-MB-231 cells were seeded in a 6-well plate and incubated for 24 h. Next, the cells were treated with ampelopsin E (7.5–30 μM) and further incubated for 72 h. The untreated cells were included as a control. The image was captured at 0, 24, 48 and 72 h at the same spot under an inverted microscope (Olympus, Japan).

### Determination of cell cycle arrest

MDA-MB-231 cells were seeded at 800,000 cells per 75 cm^2^ flask. Next, the cells were treated with ampelopsin E (7.5–30 μM). The untreated cells were also included as a control. The cells were incubated for 24, 48 and 72 h. Next, the cells were trypsinized, washed twice with ice cold PBS and collected. The cell pellet was fixed with ice-cold 70 % ethanol and stored at −20 °C. After a week, the cells were washed once again with ice-cold PBS. The cell pellet was mixed up with 250 μL Guava cell cycle reagents (EMD, Milliport Corporation, Hayward) and 300 μL PBS. The population of cells in each phase was measured by a flow cytometer (FACSCalibur, Becton Dickinson, USA). The results were analysed by using ModFit LT™ software.

### Determination of mode of cell death

Human Annexin V/PI Apoptosis Detection Kit I (BD Biosciences Pharmingen, Franklin Lakes, NJ, USA) was used to determine the mode of cell death induced by ampelopsin E. Briefly, the MDA-MB-231 cells were seeded at 150,000 cells per well in a 6-well plate. Next, the cells were treated with ampelopsin E (7.5–30 μM). The untreated cells were also included as a control. The cells were harvested and washed with 2 mL of ice-cold PBS. Subsequently, the samples were mixed with 100 μL of 4X binding buffer on ice. Next, in the dark, 5 μL annexin V and 5 μL of propidium iodide (1 mg/mL) were added with 15 min of incubation on ice. Next, 400 μL of 4X binding buffer was added to dilute the samples. The cells were analyzed by using FACS Calibur flow cytometer (Becton Dickinson, USA). The results were analysed by using FlowJo version 8.6 (Treestar Inc., San Carlos, CA).

### Statistical analysis

All values were expressed as mean ± standard deviation (SD). The data were analyzed by one-way analysis of variance (ANOVA) followed by Dunnet using Statistical Package for Social Science (SPSS) version 21.0. Probability of *p* < 0.05 was considered statistically significant.

## Results

### Ampelopsin E was cytotoxic and inhibited growth of MDA-MB-231 cells

From Table 1, ampelopsin E, Malaysianol A and flexuosol A were cytotoxic towards MDA-MB-231 cells at 14.5, 23 and 27.5 μM, while ampelopsin E was cytotoxic towards MCF-7 cells at 29.5 μM in a time- and concentration-dependent. Tamoxifen and doxorubicin were cytotoxic towards both MDA-MB-231 at 7 and 0.4 μM, respectively, and MCF-7 at 11.5 and 0.21 μM, respectively. Treatment with ampelopsin E at 15–30 μM resulted in significant reduction (*p* < 0.05) in the cell viability.

**Table 1:** Cytotoxicity of oligostilbenoid compounds isolated from *Dryobalanops* towards cancer cell lines and non-tumorigenic cell lines at 72 h reflected by the IC_50_ values as determined by MTT assay

	IC_50_ (μM)								
Compound	Flexuosol A	Nepalensinol E	Laevifonol	Ampelopsin F	Ampelopsin E	Malaysianol A	Malaysianol D	Tamoxifen (Positive control)	Doxorubicin
Cell line									
MDA-MB-231	27.5 ± 3.54	>30	>30	>30	14.5 ± 0.71	23 ± 4.24	>30	7 ± 1.41	0.4 ± 0
MCF-7	>30	>30	>30	>30	29.5 ± 0.71	>30	>30	11.5 ± 3.54	0.21 ± 0.27
HT-29	>30	>30	>30	>30	>30	N/A	N/A	N/A	N/A
A-549	>30	>30	>30	>30	>30	N/A	N/A	N/A	N/A
HeLa	>30	>30	>30	>30	>30	N/A	N/A	N/A	N/A
3T3	>30	>30	>30	>30	>30	N/A	N/A	N/A	N/A
MCF10A	>30	N/A	N/A	N/A	>30	>30	>30	N/A	>10

Each data point represents the mean of two independent experiments ± SD

**Fig. 1:**
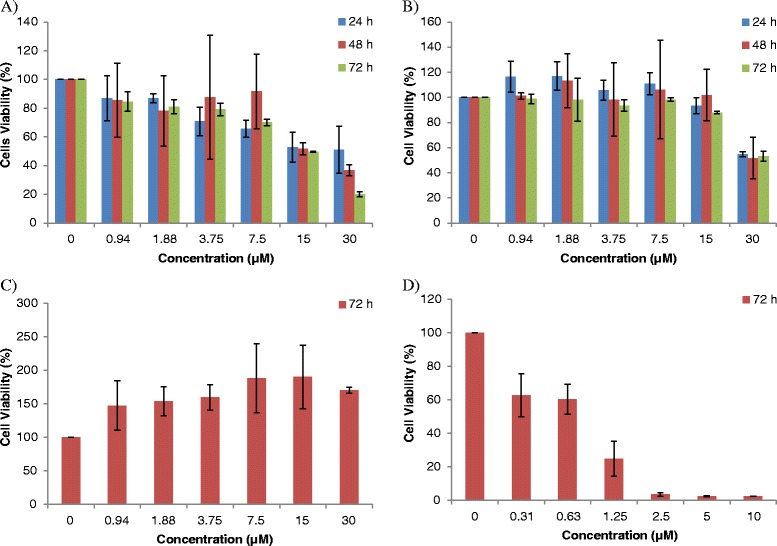
Effects of ampelopsin E on the viability of **a** MDA-MB-231, **b** MCF-7 and **c** MCF10A as determined by MTT assay. Antiproliferative effect of ampelopsin E was high compared to reference drug, doxorubibin on MDA-MB-231 cell line (**d**). The data are represented as mean percentage of viable cells ± SD of two replicates in two independent tests

The IC_50_ values of ampelopsin E towards MDA-MB-231 cells at 24, 48 and 72 h were 30 ± 0.13, 16 ± 2.01 and 14.5 ± 0.71 μM, respectively (Fig. 1a). The IC_50_ value of ampelopsin E towards MCF-7 cells at 72 h was 29.5 ± 0.71 μM (Table 1B). Ampelopsin E was less cytotoxic towards MCF-10A normal breast epithelial cells with IC_50_ more than 30 μM (Fig. 1c). The IC_50_ of doxorubicin (positive control) towards MDA-MB-231 cells at 72 h was 0.4 ± 0 μM (Fig. 1d). Based on the cytotoxic effect, 7.5, 15 and 30 μM of ampelopsin E, and incubation time of 24 and 48 h were selected for further analysis.

### The morphological changes of MDA-MB-231 cells treated with ampelopsin E

**Fig. 2:**
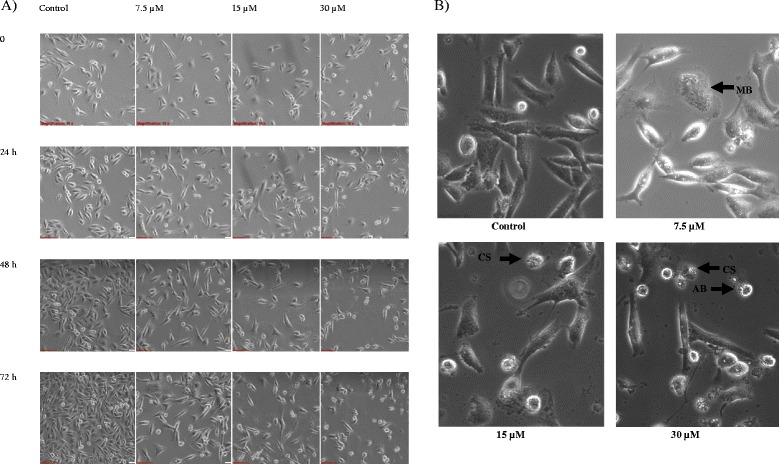
**a** Morphological changes of ampelopsin E-treated MDA-MB-231 cells observed under an inverted light microscope (100 × magnification). Cell population decreased with the increase in the compound concentration. **b** The ampelopsin E-treated MDA-MB-231 cells showed the features of apoptosis such as membrane blebbing (MB), cellular shrinkage (CS) and formation of apoptotic bodies (AB) at 48 to 72 h (400× magnification)

From Fig. 2a, the number of MDA-MB-231 cells reduced at 15 and 30 μM of ampelopsin E at 48 to 72 h. Cell detachment, cell shrinkage and formation apoptotic bodies were observed at 48 and 72 h in MDA-MB-231 cells treated with 30 μM of ampelopsin E (Fig. 2b). At 7.5 μM of ampelopsin E, membrane blebbing was noted while at 15 μM of the compound, cell shrinkage was observed. Nevertheless, the cell number increased over time.

### Ampelopsin E induced G_2_/M arrest in MDA-MB-231 cells

**Fig. 3:**
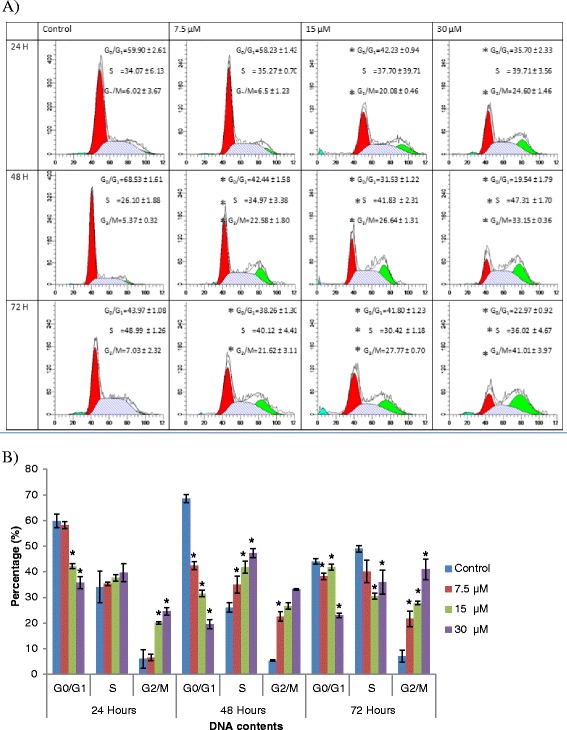
Cell cycle profile of MDA-MB-231 cells treated with ampelopsin E (**a** and **b**). Each data point represents the mean of three independent experiments ± SD. *significantly different from the control (*p* < 0.05)

The cell cycle arrest of MDA-MB-231 cells by ampelopsin E was time- and concentration-dependents (Fig. 3). At 24, 48 and 72 h, an increase in G_2_/M population at 15 and 30 μM of ampelopsin E was noted (*p* < 0.05). In addition, at 7.5, 15 and 30 μM of ampelopsin E at 72 h, increase in the number of cells in S phase compared to control, accompanied by a decline in G_0_/G_1_ phase cell population was observed (*p* < 0.05).

### Ampelopsin E induced apoptosis in MDA-MB-231 cells

**Fig. 4:**
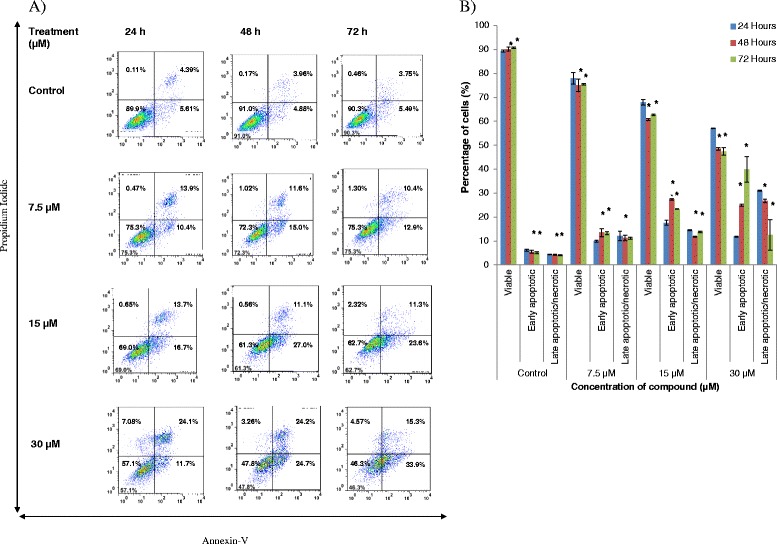
The percentage of viable, apoptotic and necrotic/secondary necrotic cells of untreated andampelopsin E-treated MDA-MB-231 cells for 24 and 48 h as determined by flow cytometer. **a** and **b** These are from representative experiments carried out at least three times. The percentage of viable cells were represented by lower left quadrant (Annexin-V^−^/PI^−^); the percentage of early apoptotic and necrotic/secondary necrotic cells were represented by the lower right (Annexin-V^+^/PI^−^) and upper (PI^+^) quadrants, respectively. Each data point represents the mean of three independent experiments ± SD. *significantly different from the control (*p* < 0.05)

Ampelopsin E induced apoptosis in MDA-MB-231 cells. At 24 h, the percentage of viable cells decreased from 75.3 % at 7.5 μM, 69.0 % at 15 μM and 57.1 % at 30 μM of ampelopsin E compared to 89.9 % in the control (*p* < 0.05). From Fig. 4, the percentage of early apoptotic cells increased in a time- and concentration-dependent manner. At 72 h, the percentage of early apoptotic increased from 12.9 % at 7.5 μM, 23.6 % at 15 μM and 33.9 % at 30 μM of ampelopsin E compared to 5.49 % in the control (*p* < 0.05).

## Discussion

Previously, from a study done by Fisher et al. [7], the use of tamoxifen should continue to treat breast cancer because of net benefit greatly outweighs the risk (increase severity of cancer). However, plants also are a good and potential source of anticancer agents especially to replace the drug such as tamoxifen with some adverse effects. For instance, vindesine from *Catharanthus roseus* [5], taxol from *Taxus brevifolia* [20], beta-lapachone from *Tabebuia avellanedae* [23] and genistein from *Glycin max* [19] have been employed clinically to treat breast cancer patients.

In this study, several compounds isolated from *Dryobalanops sp* (ampelopsin E, ampelopsin F, flexuosol A, laevifonol, Malaysianol A, Malaysianol D and nepalensinol E) were tested towards various cell lines (MDA-MB-231, MCF-7, HT-29, A-549, HeLa, 3 T3 and MCF10A). Based on the IC_50_ values, ampelopsin E was the most cytotoxic towards MDA-MB-231 cells at 14.5 μM compared to MCF-7, HT-29, A-549, HeLa, 3 T3 and MCF10A. Ampelopsin E, Malaysianol A and Malaysianol D are stereoisomer with similar basic skeleton [34]. Based on Wibowo et al. [34], Malaysianol A and ampelopsin E exhibited almost similar cytotoxic effects, but Malaysianol D showed lower activity towards MCF-7 and A-549. The free stilbene skeleton of flexuosol A has no contribution at all. It is suggested that the polarity and stereochemistry of oligostilbenoid constituents are important factors influencing their cytotoxicity. According to the National Cancer Institute (NCI) guidelines, ampelopsin E can be considered as a great candidate to be developed as an anticancer agent for breast cancer as the IC_50_ value was less than 15 μM towards MDA-MB-231 cells after treatment for 72 h. Ampelopsin E was most potent towards the triple negative hormone receptor breast cancer cell line, MDA-MB-231. Some proteins may be related to the mechanism of drug resistance in MDA-MB-231 cells especially in DNA recombination, cell cycle, complex assembly, cytoskeleton organization, transport and negative regulation of cell death [30]. Therefore, it is deduced that ampelopsin E is a good candidate for treating drug resistant breast cancer. Ampelopsin E was cytotoxic towards the cells in a time- and concentration-dependent manner. Ampelopsin E was less cytotoxic towards normal cells (3 T3 and MCF10A) compared to the cancer cells. This suggests selectivity of ampelopsin E towards cancer cells.

Wibowo et al. [34] reported that ampelopsin E from *Dryobalanops beccarii* was most cytotoxic towards MCF-7 (IC_50_ = 20.98 μM). Nevertheless, in this study, ampelopsin E was the most cytotoxic towards MDA-MB-231 (IC_50_ = 14.5 μM). The IC_50_ towards MCF-7 was 29.5 μM. The difference is believed due to batch-to-batch variation of the cells.

The ability of ampelopsin E to induce apoptosis in MDA-MB-231 suggests that it has the potential as a new candidate of anticancer agent against independent-hormonal breast cancer. Plant extracts or compounds such as phenolics, flavonoids, tea polyphenols, alkaloids, polysaccharides and glycoproteins, lectins, tannins and lignins, terpenoids, isoprenoids and quinones [32] have been reported to selectively induce apoptosis in neoplastic cells instead of normal cells.

Ampelopsin E inhibited the growth of MDA-MB-231 cells in a time- and concentration- dependent manner. Several features of apoptosis were noted at 48 and 72 h such as membrane blebbing, cellular shrinkage and formation of apoptotic bodies [26]. There are several dynamic cellular categories that lead to cell death based on their biochemical and morphological characteristics, which are apoptosis, autophagy, necrosis, mitotic catastrophe and senescence (in cancer therapy context). Treatments that can increase the rate of apoptosis could be used to manage cancers [10] because apoptosis is a self-destructive mode that involves only the individual cells and will not cause inflammation especially to the neighboring cells [16].

Inhibition of cell growth at 15 and 30 μM of ampelopsin E between 48 and 72 h was due to cell cycle arrest at G_2_/M phase as being confirmed by flow cytometer analysis evidenced by a significant increased population in G_2_/M phase with decrease cell number of S phase. The major regulator of G_2_ to M transition is the M-phase-promoting factor (MPF) such as catalytic subunit cdc2 and regulatory subunit cyclin B1 [37]. From the study done by Huang et al. [12], dihydromyricetin isolated from *Ampelopsis grossedentata* induced G_2_/M phase cell cycle arrest through Chk1/Chk2/Cdc25C pathway. The important key roles in this pathway are the formation of CDK1/cyclin B1 and CDK1/cyclin A complexes.

Evasion of apoptosis is the requirement for cancer cells to survive [14]. Thus, understanding the apoptosis signaling pathways and the mechanism of resistance towards apoptosis are vital to manage cancer [14]. Apoptosis is a programmed cell death, which is important in animal development, tissue homeostasis and other disease process [36] as the common response to cell stress induced by physiological changes, drugs, agents or toxins [10]. There are two types of apoptotic pathway, which are extrinsic and intrinsic pathway. In the extrinsic pathway, the commonly involved proteins are TNF-alpha and NF-kappaB. TNF-α will give response towards any environmental changes such as injury and inflammation that result in apoptosis [4]. TNF-α activation can promote NF-ƙB to participate in development, survival, proliferation and metastases in cancer [28]. In the intrinsic pathway, apoptosis involves mitochondria dysfunction of Bcl-2 family of proteins. Bcl-2 family includes pro-apoptotic (Bax, Bak, Bad and Bim) and anti-apoptotic (Bcl-2, Bcl-xL and Mcl-1) [36].

The induction of apoptosis by ampelopsin E in MDA-MB-231 cells was confirmed by annexin V/propidium iodide (annV/PI). Generally in in vivo, the apoptotic bodies are engulfed by phagocytocytes such as neutrophils and macrophages. However, in vitro system lack of these phagocytes, the apoptotic bodies membrane will rupture and accessible to PI dye. Therefore, the secondary necrotic cells are stained by annexin V and PI [8]. From the principles, apoptotic and necrotic cells can be differentiated.

## Conclusion

In conclusion, ampelopsin E was most cytotoxic towards MDA-MB-231 cells. Ampelopsin E caused G_2_/M phase cell cycle arrest and induced apoptosis in the cells. Thus, ampelopsin E has potential to be developed as an anti-breast cancer agent, especially for the independent-hormonal breast cancer.

## Abbreviations

*BRCA1*: Breast cancer 1 gene
Cdc25C: Cell division cycle 25C
Chk1: checkpoint kinase 1
Chk2: Checkpoint kinase 2
DMSO: Dimethyl sulfoxide
ER: Estrogen receptor
HER: Human epidermal growth factor receptor 2
MTT: 3-[4, 5-dimethylthiazol-2-yl]-2,5 diphenyltetrazolium bromide
NF-ƙB: Nuclear factor kappa beta
PBS: Phosphate-buffered saline
PI: Propidium iodide
PR: Progesterone receptor
TNBC: Triple negative breast cancer
TNF-α: Tumor necrosis factor
